# Nafamostat mesylate versus regional citrate anticoagulation for chronic hemodialysis in patients at high risk of bleeding: a single-center, retrospective study

**DOI:** 10.1080/0886022X.2025.2464830

**Published:** 2025-02-20

**Authors:** Jiangtao Li, Lirui Wang, Yuqiu Lu, Ying Zhou, Yue Chen

**Affiliations:** Department of Nephrology, Tongji University School of Medicine, Tongji Hospital, Shanghai, China

**Keywords:** Hemodialysis, anticoagulation, nafamostat mesylate, citrate

## Abstract

**Introduction:**

For hemodialysis patients at high risk of bleeding, a regional anticoagulant can be used, such as citrate or nafamostat mesylate (NM). The objective of this study was to evaluate NM as an alternative to citrate for anticoagulation in hemodialysis patients at high risk of bleeding.

**Methods:**

This retrospective single-center study included consecutive patients in our dialysis center treated with either citrate or NM anticoagulation for hemodialysis from January 2022 to December 2023.The primary outcome was major clotting, defined as premature dialysis due to extracorporeal circuit clotting. The secondary outcome was the incidence of a major bleeding episode during or after hemodialysis.

**Results:**

In total, 651hemodialysis sessions were performed in 196 patients and were compared (289 citrate and 362 NM anticoagulation). A lower number of premature dialysis due to clotting occurred in the NM sessions compared to citrate sessions (0.84% vs.5.19%, *p* = 0.001). NM was associated with a lower risk of major clotting compared with citrate during treatment (OR:0.063; CI: 0.008-0.475; *p* = 0.007). Regarding second outcome, no more major bleeding events related to NM occurred compared to citrate.

**Conclusion:**

Among hemodialysis patients with high risk of bleeding, anticoagulation with NM, compared with citrate anticoagulation, provided relatively better efficacy, with no bleeding increment. NM is a valid alternative to citrate for hemodialysis patients at high risk of bleeding.

## Introduction

Hemodialysis is a life-supporting therapy for patients with chronic renal failure. Successful hemodialysis depends on adequate anticoagulation. Anticoagulation maintains the flow of blood in the extracorporeal circuit and prevents thromboembolic events during hemodialysis. For the patients at high risk of bleeding, the optimal strategy is a method that avoids systemic anticoagulation but reliably prevents clotting of the circuit [1].

Citrate is a preferred regimen for regional anticoagulation because its anticoagulation effect is limited to the extracorporeal circuit [2]. The international gold standard of (RCA) using calcium-free dialysates can achieve full anticoagulation effects by using a calcium-free dialysate [3]. In real-world practice, however, the majority of medical centers in China, had to make a compromise of using commercially available calcium-containing dialysates instead of calcium-free ones due to their scarcity [4–6]. Nevertheless, RCA using calcium-containing dialysates resulted in significantly worse anticoagulation of dialyzer and venous bubble trap [7].

Nafamostat mesylate (NM) is a serine protease inhibitor that has inhibitory effects on various coagulation factors and platelet activation [8,9]. NM in the blood can be rapidly degraded by hepatic carboxylesterase and cleared by hemodialysis [10,11]. With a short half-life of 8 min in the blood, NM is used as a regional anticoagulant during hemodialysis in patients at high risk of bleeding in Japan [12–14]. NM has now been included in the national medical insurance catalog, making it significantly cheaper than citrate, which must be purchased out-of-pocket. For these reasons, in China, NM is increasingly used for hemodialysis patients at high risk of bleeding.

Although citrate using calcium-containing dialysates and NM are both used for hemodialysis in patients at high risk of bleeding, no studies have compared them so far. This study aimed to compare the efficacy and safety of these two anticoagulants for hemodialysis to guide clinical use better.

## Materials and methods

### Research design and objects

**Figure 1.**
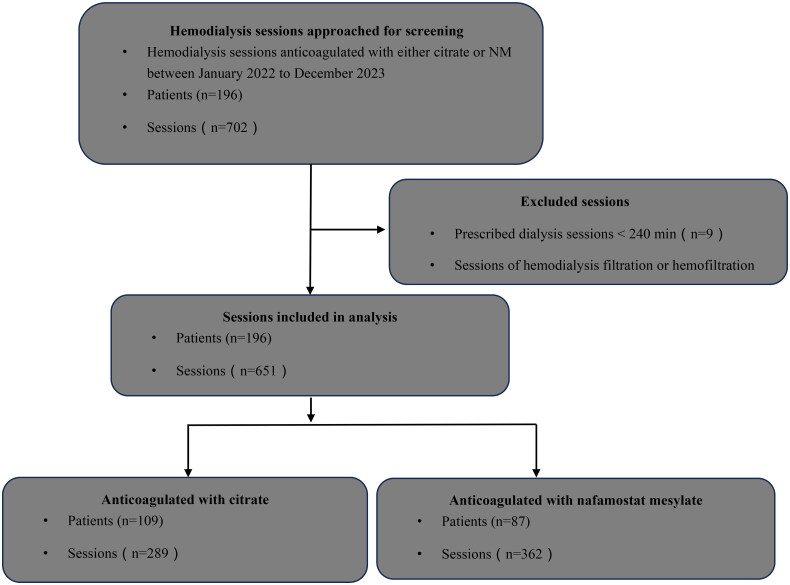
Flow chart for study inclusion/exclusion of patients.

This was a retrospective observational study from a single center. Hemodialysis patients at high risk of bleeding who anticoagulated with either citrate or NM between January 2022 to December 2023 were considered as candidates. High risk of bleeding was defined as follows [15]: active bleeding has now ceased for no more than 3 days; surgical or traumatic wounds within the past 3 days; or acute dialysis *via* an intravenous catheter. Prescribed dialysis sessions less than 240 min and sessions of hemodialysis filtration or hemofiltration models were excluded. The study was approved by the local Ethics Committee and was conducted in agreement with the Declaration of Helsinki. The need for informed consent was waived. Flow chart for study inclusion/exclusion of patients was shown in Figure 1.

### HD delivery

During the dialysis treatment, conventional hemodialysis machines were mainly used, including Fresenius (4008S type, Fresenius Medical Care), Gambro (AK200S type, Gambro Lundia AB), and polysulfone membrane dialyzers adopting Fresenius FX8 (Fresenius Medical Care) and Gambro 14 L (Gambro Dialysatoren GmbH, Hechingen, Germany). Dialysate was composed of sodium, 140 mmol/L; bicarbonate, 34 mmol/L; calcium, 1.5 mmol/L; and magnesium, 0.5 mmol/L. The dialysate flow rate was the same for all patients, which was 500 mL/minute. The blood flow rate and ultrafiltration rate were set and adjusted according to therapeutic needs. Dialysis access was *via* an arteriovenous fistula or a central venous catheter.

### NM protocol

NM (Durui Pharmaceutical Co., LTD, Jiangsu, China) is dissolved in 5% glucose injection at a dose of 20-40 mg per hour through an anticoagulant infusion line continuously, with moderate decreases or increases depending on clinical indications.

### Citrate protocol

A sterile solution of trisodium citrate (4% TSC; 136 mmol/L; QinShanLiKang Pharmaceutical CO. LTD., SiChuan, China) was continuously infused into the arterial line at a rate equal to 2% of dialyzer blood flow. Calcium supplementation was achieved with the diffusion of calcium contained in the dialysate. Ionized calcium was monitored at least one hour after and at the end of a session. When the patient’s ionized calcium level was lower than 0.90 mmol/L, 10% calcium gluconate solution was infused.

### Data collection

Patient and hemodialysis characteristics were extracted from the patient’s electronic medical record. Patient characteristics included age, sex, primary kidney disease requiring hemodialysis, type of vascular access, anticoagulation type (citrate or NM) and contraindication to heparin anticoagulation defined as high risk of bleeding. Hemodialysis characteristics included vascular accesses, blood flows and ultrafiltration rates.

### Outcome measures

The primary outcome was major clotting, defined as premature dialysis due to extracorporeal circuit clotting. The secondary outcomes were as follows: (1) incidence of a major bleeding episode during hemodialysis, (2) shortened dialysis time due to clotting, (3) adverse drug reactions associated with anticoagulants administration. Major bleeding defined as new or worsened bleeding that required hospitalization or transfusion, bleeding into either the central nervous system or the retroperitoneuma, or bleeding that resulted in death [16].

### Statistics

Parametric and nonparametric parameters are expressed as mean ± SD and median and interquartile range, respectively. Categorical variables were described by counts and percentages. Statistical analysis was performed using the independent-sample *t* test, the χ2 test, or Fisher’s exact test, depending on the data type. *P*-values < 0.05 were considered statistically significant. To determine if NM was associated with a lower risk of major clotting compared with citrate, we used generalized estimating equation analysis with a logistic link function with an unstructured correlation matrix (up to 100 iterations) [17]. Adjustment for anticoagulation type, type of vascular access, mean blood flow and ultrafiltration rates was performed. IBM SPSS Statistics for Windows (version 28.0. Armonk, NY: IBM Corp) was used for statistical analysis.

## Results

### Patient demographics

Patient’s relevant characteristics are indicated in Table 1. In summary, 196 patients were enrolled in this study. Among these patients, 109 (55.61%) anticoagulated with citrate, whereas 87 (44.39%) treated with NM. There were no significant differences in baseline characteristics between the citrate group and NM group.

**Table 1.** Patients characteristics.

	RCA group (*n* = 109)	NM group (*n* = 87)	*p*-value
Female, n (%)	41 (37.61%)	26 (29.55%)	0.274
Age, median (IQR), years	66.26 ± 14.50	63.39 ± 12.39	0.143
Primary kidney disease, n (%)			0.945
Chronic glomerulonephritis	47 (43.12%)	40 (45.98%)	
Diabetic nephropathy	36 (33.02%)	28 (32.18%)	
Hypertensive nephropathy	11 (10.09%)	10 (11.49%)	
Polycystic kidney disease	9 (8.26%)	6 (6.89%)	
Other secondary kidney diseases	6 (5.51%)	3 (3.46%)	
Drug use before treatment			
Oral anticoagulants	5 (4.59%)	2 (2.30%)	0.466
Antiplatelet aggregation drug	82 (75.23%)	64 (73.56%)	0.869
Erythropoietin stimulating agents	104 (95.41%)	84 (96.55%)	1.000
Proton pump inhibitors	19 (17.43%)	13 (14.94%)	0.700
NSAID	11 (10.09%)	3 (3.45%)	0.094
Glucocorticoids	2 (1.83%)	1 (1.15%)	1.000
Reasons for high bleeding risk, n (%)			0.471
Tunneled hemodialysis catheter placement	60 (55.05%)	39 (44.83%)	
GI bleeding	30 (27.52%)	29 (33.33%)	
Renal cyst bleeding	9 (8.26%)	13 (14.94%)	
Arteriovenous access placement	5 (4.58%)	4 (4.60%)	
Other invasive procedure	4 (3.67%)	2 (2.30%)	
Central nervous system trauma	1 (0.92%)	0 (0%)	
Dialysis access (patients %)			0.563
Arteriovenous fistula	46 (42.20%)	41 (47.13%)	
TCC	63 (57.80%)	46 (52.87%)	
Hemoglobin (g/L)	90.17 ± 20.54	93.17 ± 23.71	0.343
Blood platelets (10^9^/L)	171.15 ± 81.85	167.21 ± 63.37	0.711
Number of treatments	289	362	
Median (IQR)	2	2	0.087
Minimum/maximum	1/10	1/19	

RCA: regional citrate anticoagulation; NM: nafamostat mesylate; AVF: arteriovenous fistula; TCC: tunneled cuffed catheter; GI: gastrointestinal.

### Characteristics of dialysis sessions

Operational characteristics are listed in Table 2. A total of 651 hemodialysis sessions were performed during the study period, including 289 sessions using citrate and 362 sessions using NM anticoagulation. There were statistically significant differences (*p* < 0.001) in vascular access and blood flow between the citrate sessions and the NM sessions. Other baseline characteristics, such as dialysate flows and ultrafiltration rates, showed no statistical significance (*p* > 0.05; Table 2).

**Table 2.** Hemodialysis characteristics.

	RCA group (*n* = 289)	NM group (*n* = 362)	*p*-value
Access (AVF/TCC)	125/164	211/151	<0.001
QB (mL/min)	200.40 ± 10.68	218.82 ± 24.10	<0.001
Qdialysate (mL/min)	500	500	
Quf(ml/h)	1905.33 ± 522.33	1229.62 ± 551.31	0.337
Early dialysis termination due to clotting, n (%)	15 (5.19%)	3 (0.83%)	0.001
Shortened dialysis time due to clotting(min)	30 (30,30)	30 (30,30)	0.083
Clotting site, n (%)			0.655
Dialyzer	1 (0.35%)	0 (0%)	
Venous expansion chamber	14 (4.84%)	3 (0.83%)	
Arterial expansion chamber	0 (0%)	0 (0%)	

AVF: arteriovenous fistula; TCC: tunneled cuffed catheter; QB: blood flow; Qdialysate: dialysate flow; Quf: ultrafiltration rates.

### Study outcomes

#### Primary outcomes

Major clotting occurred in 18 of 651sessions, including 15 of 289 citrate sessions and 3 of 362 NM sessions. The occurrence of major clotting was more frequent with citrate compared to NM (5.19% vs. 0.84%, *p* = 0.001) (Table 2).

When controlling for possible confounding variables, such as type of vascular access, mean blood flow and ultrafiltration rates, NM was associated with a lower risk of major clotting compared with citrate during treatment (OR:0.063; CI: 0.008-0.475; *p* = 0.007). (Table 3).

**Table 3.** GEE Analysis of the impact of different anticoagulants on the success rate of HD.

Variable	Univariable (OR, [95% CI])	p-value	Multivariable (aOR, [95% CI])	p-value
RCA	Ref		Ref	
NM	0.147 (0.042-0.505)	0.002	0.063 (0.008-0.475)	0.007
AVF	Ref		Ref	
TCC	1.237 (0.456-3.355)	0.676	1.954 (0.585-6.527)	0.276
QB	0.978 (0.943-1.013)	0.217	0.996 (0.939-1.056)	0.881
Quf	1.000 (0.999-1.000)	0.201	0.998 (0.995-1.000)	0.060

RCA: regional citrate anticoagulation; NM: nafamostat mesylate; AVF: arteriovenous fistula; TCC: tunneled cuffed catheter; QB: blood flow; Quf: ultrafiltration rates; OR: odds ratio; aOR: adjusted odds ratio.

#### Secondary outcomes

##### Major bleeding

Reasons for bleeding risk were listed as patients on the day of the first hemodialysis (Table 1). We did not observe any new or worsened bleeding that required hospitalization or transfusion, bleeding into a critical organ or space, or bleeding that resulted in death.

##### Shortened dialysis time due to clotting

Shortened dialysis time due to clotting was not significantly different in NM and citrate groups (Table 2).

##### Adverse drug reactions

The vital signs of patients in the two groups were stable during treatment. In the citrate group, none of the patients experienced symptoms of hypocalcemia. No patients experienced serious adverse drug reactions (e.g., severe anaphylaxis, eosinophilia, agranulocytosis and myelosuppression) associated with NM administration.

## Discussion

No study has so far synthetically compared the efficacy and safety of citrate and NM in hemodialysis patients. Our data suggest that, when compared with citrate using calcium-containing dialysate, the use of NM during hemodialysis results in significantly better anticoagulation with no bleeding increment.

Before NM is included in the medical insurance catalog in 2022, a simplified RCA (S-RCA) method using calcium-containing dialysate has been preferentially selected at our center for hemodialysis patients at high risk of bleeding. Despite a long tradition, high volume and expansion of S-RCA use, anticoagulation in our center is not always achievable. When this method is used, as in our current study, the premature termination rate of hemodialysis due to severe clotting was 5.19%. Wang et al. reported the use of conventional calcium-containing dialysate (1.25-1.5 mmol/L) and the total clotting in their study was 16.66% [6]. In another smaller series of 25 dialysis sessions with calcium-containing dialysate, 4 of 25 HD procedures (16%) were prematurely terminated due to threatening dialyzer clotting [18]. From these data, it seems that RCA with calcium-containing dialysate provides a somewhat poorer antithrombotic effect.

NM is a serine protease inhibitor, and has such activities as anti-coagulant effect, anti-fibrinolytic activity, and anti-platelet actions. Due to a short half-life of eight minutes, it has recently been used prevalently during hemodialysis (HD) in patients with a high risk of bleeding. Several studies comparing NM with heparin in hemodialysis patients have reported better efficacy during NM anticoagulation [13,14].

There are two retrospective studies of NM vs. citrate in CRRT population and both showed comparable efficacy [19,20]. Those results, however, may not be applicable for patients undergoing hemodialysis due to differences in duration of therapy and the complexity of the circuit. In our current study, a lower number of premature clotting occurred (0.84%) in the NM sessions compared to the citrate sessions (5.19%). When controlling for possible confounding variables, such as type of vascular access, mean blood flow and ultrafiltration rates, NM was associated with a lower risk of major clotting compared with citrate during treatment. It may be concluded from our data that hemodialysis with NM is superior to citrate using calcium-containing dialysate as far as major clotting is concerned.

No major bleeding and serious adverse reactions (e.g., severe anaphylaxis, eosinophilia, agranulocytosis, and myelosuppression) associated with NM administration was recorded in this study. It should be emphasized that we did not monitor the change of hemotologic parameters. The subjective assessment by us may not correspond to blood lose quantifiable by change in hemotologic parameters. As such, a careful and intensive follow-up, not for visible evidence of bleeding but also for reduction of hemoglobin that indicate bleeding complication, is needed.

There are several limitations to this study. First, the analysis was retrospective, and some data baselines were different. The choice of anticoagulation was at the discretion of the treating physicians. Second, we did not observe the effectiveness of the hemodialysis filtration or hemofiltration models, which are also major patterns of blood purification in clinical practice. Third, this study was generated using polysulfone membranes. Polysulfone membranes, which is less effective in adsorbing NM [21], might affect systemic coagulation function. Therefore, monitoring indicators of hemostasis and coagulation function are necessary. Finally, the use of calcium-containing dialysate for RCA deviated from the international gold standard of calcium-free RCA. This may lead to underestimating RCA’s true efficacy, limiting the applicability of the findings to other regions.

## Conclusion

Among patients at high risk of bleeding receiving hemodialysis, anticoagulation with NM, compared with citrate anticoagulation, provided relatively better efficacy, with no bleeding increment. NM is a valid alternative to citrate for hemodialysis patients at high risk of bleeding.

## Supplementary Material

Ethics.pdf

## Data Availability

The data sets used and/or analyzed during the current study are available from the corresponding author on reasonable request.
