# Supplementary material for: Nafamostat mesylate versus regional citrate anticoagulation for chronic hemodialysis in patients at high risk of bleeding: a single-center, retrospective study
Source: Ren Fail. 2025 Feb 20;47(1):2464830. doi: 10.1080/0886022X.2025.2464830 (PMC11849013; doi:10.1080/0886022X.2025.2464830)
Supplement: Ethics.pdf [file IRNF_A_2464830_SM1241.pdf]

海市同济医院（同济大学附属同济医院）伦理委员会批准函

ShanghaiTongji Hospital （Tongji Hospital of Tongji University）

Ethics Committee Approval Letter

批件号	（同）伦审第（K-2024-043）号			
项目名称	甲磺酸奈莫司他与枸橼酸钠在高危出血风险血透患者中抗凝有效性和安全性的对比研究			
申办方	上海市同济医院			
审查类别	☑初始审查			
	☐复审<br>（仅勾选此项需填）	初始审查日期	审查方式	审查结果
组长单位	上海市同济医院			
主要参加单位	无			
承担科室	肾内科	主要研究者	李江涛	
审查日期	2024.8.15	审查地点	同济医院教学楼 101 会议室	

审查情况				
审查方式	应到人数	实到人数	投票人数	利益冲突回避人员
☐会议审查				
☑快速审查	不适用	不适用	不适用	无

审查决定：

☑同意　　请遵循已经伦理委员会批准的方案执行，伦理批准时间为批件签发日期。

必要修改后同意（ ☐作必要的修改后同意　☐作必要的修改后重审）

请按审批意见逐条修改或陈述，申请伦理委员会复审，经批准同意后方可开展。

☐不同意

☐暂停或终止已批准的项目

审查文件：

见附件

审查频率为研究首批准之日起：	12 个月
批件有效期为研究首批准之日起：	3 年

主任或副主任委员签字　（签名）　签发日期 2024.8.15

伦理委员会　（盖章）

声明：请仔细阅读

上海市同济医院（同济大学附属同济医院）伦理委员会的职责、人员组成、操作规范和记录遵循 CFDA《药物临床试验质量管理规范（2020)》，CFDA《药物临床试验伦理审查工作指导原则（2010)》、《医疗器械临床试验质量管理规范（2022)》、《体外诊断试剂临床试验技术指导原则（2021)》、《中华人民共和国人类遗传资源管理条例（2019)》、卫生部《涉及人的生物医学研究伦理审查办法（2016)》、《赫尔辛基宣言（2013)》、《人体生物医学研究国际道德指南（2016)》和 ICH-GCP 的伦理原则并遵守中国相关法律和法规。
